# Lignin-Based Hollow Nanoparticles for Controlled Drug Delivery: Grafting Preparation Using β-Cyclodextrin/Enzymatic-Hydrolysis Lignin

**DOI:** 10.3390/nano9070997

**Published:** 2019-07-11

**Authors:** Yu Zhou, Yanming Han, Gaiyun Li, Sheng Yang, Fuxiang Chu

**Affiliations:** 1 Research Institute of Wood Industry, Chinese Academy of Forestry, Xiangshan Road, Beijing 100089, China; 2 School of Chemistry and Chemical Engineering, Yancheng Institute of Technology, Yancheng City 224051, China

**Keywords:** lignin, β-cyclodextrin, hollow nanoparticles, hydroxycamptothecin, sustained release

## Abstract

Due to its abundance, degradability, and low toxicity, lignin is a promising raw material for the preparation of nanomaterials. However, efficient encapsulation using lignin-nanomaterial for sustained-release medications remains a challenge. This study involves grafting β-cyclodextrin (β-CD), with a hollow toroidal structure, onto the enzymatic-hydrolysis lignin (EHL) to form CD-EHL. The modified lignin was next used to prepare hollow nanoparticles (LHNPs) via self-assembly to encapsulate the antitumor drug hydroxycamptothecin (HCPT). The results indicated that β-CD improved the network structure of modified lignin molecules. Moreover, LHNPs that self-assembled using CD-EHL had an increased specific surface area and greater porosity, and exhibited a spherical hollow structure and stability in phosphate-buffered saline. The drug loading and encapsulation efficiency of HCPT were 70.6 ± 9% and 22.02 ± 2%, respectively. An *in vitro* study showed that lignin-based nanoparticles have low toxicity, and the modified LHNPs demonstrated a good sustained-release capability. This study broadened the potential application of lignin as a renewable biomass material.

## 1. Introduction

Lignin is an amorphous polyphenol and the major constituent of plant cell walls. Its abundance in the biosphere is second only to cellulose [1]. Lignin has become a popular biomaterial, with antibacterial and antioxidant properties, non-cytotoxicity, and biodegradability [2]. The production of enzymatic-hydrolysis lignin (EHL) as biorefinery waste has increased year by year. Additionally, numerous phenolic and alcoholic hydroxyl groups on molecular chains can be easily modified with functional groups or conjugated with new functional groups [3]. Together, these properties make lignin an attractive material for development and an important recyclable industrial by-product.

Cancer is a critical public health problem worldwide [4]. Currently, tumor treatments include surgery and chemotherapy. However, due to factors such as imperfect tumor localization and the rapid diffusion of cancer cells, tumor tissue is very difficult to excise completely. Thus, chemotherapy remains an important method for treating cancer [5,6]. Anticancer drugs can effectively inhibit the proliferation of tumor cells, thus showing good therapeutic effect. Hydroxycamptothecin (HCPT) is an alkaloid extracted from the Chinese-native *Camptotheca acuminata* Decne [7]. The solubility of HCPT is poor and it is quickly metabolized, leading to an extremely short half-life. As a dose-dependent drug, toxicity and side effects are considerable for the direct use of HCPT [8]. For these reasons, there is a strong interest in the development of HCPT delivery systems to allow controlled metabolism in sustained-release medications, improved bioavailability, and reduced toxicity and side effects. One strategy for these delivery systems is the use of hollow nanoparticles.

The ideal drug delivery system should be made of easily available material sources, have a low cost of synthesis, modifiable surfaces, great biodegradability and biocompatibility, stimulating responsiveness, and, critically, low or no toxicity [9,10]. Overall, safe and reproducible carriers are required, and biomaterials are promising candidates for use as these carriers [11]. Among biomaterials, there has been a focus on the use of lignin-based hollow nanoparticles (LHNPs) as new drug delivery platforms [12]. LHNPs have been demonstrated as capable of loading drugs [13,14,15]. However, an important requirement for a biological drug-loading material is a specific interaction with the encapsulated drugs. Previous studies have proved that β-cyclodextrin (β-CD) can control drug release via forming complexes formed with hydrophobic medications [16,17]. However, no introduction has been reported of cyclodextrin (CD) monomers with a hollow structure and a hydrophilic exterior and hydrophobic interior, to lignin molecular chains to expand the network function. The modification of EHL by β-CD expands the network structure of lignin molecules and generates specific sites to interact with medications [18].

Herein, we report a new type of amphiphilic polymer obtained by the β-CD modification of lignin, and investigated the self-assembly of the polymer to hollow particles for encapsulation of sustained-release HCPT medications. The surface properties and encapsulation efficiency of these particles as well as their ability to allow for sustained drug release were comprehensively investigated.

## 2. Materials and Methods

### 2.1. Materials

Enzymatically-hydrolyzable lignin (EHL) was acquired from Hong Kong Laihe Biotechnology Co., Ltd. (Hong Kong, China). The hydroxyl content, average molar mass, and dispersity of the EHL were 2.67 mmol/g, 1430 g/mol, and 1.22, respectively. Tetrahydrofuran (THF) of an analytical-grade purity was provided by the Beijing Chemical Reagents Co. (Beijing, China). The 3-aminopropyltriethoxysilane (APTES), mono-6-O-(p-toluenesulfonyl)-beta-cyclodextrin (6-TsO-β-CD), and potassium iodide (KI) were obtained from the Shanghai Macklin Biochemical Co., Ltd. (Shanghai, China). Hydroxycamptothecin (HCPT) was purchased from Shanghai Macklin Biochemical Co., Ltd. (Shanghai, China), with a purity rating above 99%. All chemicals were used without further treatment.

### 2.2. Modification of Lignin (A-EHL)

First, 0.5 g EHL was ultrasonicated for 5 min and then 40 mL was dried by molecular sieve in a three-necked flask under the protection of N_2_. Then, APTES (1.5 mL) was added and stirred, using a magnetic stirrer at room temperature (25 °C) for 4 h. After reaction, the THF was removed by rotary evaporation. A-EHL with amino was obtained after rinsing the material three times with diethyl ether and then the materials were finally dried in a vacuum.

### 2.3. Grafting of β-CD (CD-EHL)

First, 100 mg of A-EHL and 50 mg of 6-TsO-β-CD were ultrasonicated for 5 min with N-Dimethylacetamide (20 mL) and then dried by molecular sieve in a three-necked flask under protection of N_2_. Then, 2 mg of KI was added, followed by stirring at 1000 rpm for 6 h at 70 °C. After cooling to room temperature, the precipitate was obtained after the addition to 1 L of HCl (2 M). The precipitate was centrifuged at 6000 rpm for 4 min, the supernatant was removed, and the precipitated material was then washed 3 times with deionized water. The obtained product was stored at room temperature in a vacuum drying oven.

### 2.4. Preparation of CD-EHL Hollow Nanoparticles (CD-LHNPs)

To prepare the CD-EHL hollow nanoparticles (CD-LHNPs), 10 mg of CD-EHL was dissolved in THF to prepare a 1 mg/mL EHL–THF solution at room temperature. The solution was then stirred at 1000 rpm with a magnetic stirring bar. Next, 40 mL of water was added dropwise into the solution at a rate of 7 mL/min, inducing the self-assembly of lignin molecules into hollow nanoparticles (HNPs). The suspension liquid was stirred continuously to evaporate the THF. After 6 h, the suspension liquid was transferred to a dialysis bag (MWCO: 12,000−14,000; Spectrum Labs, San Francisco, CA, USA) which was then immersed in deionized water (that was periodically replaced), to remove the residual THF before further measurements.

### 2.5. Drug Loading

The HCPT-loaded LHNPs (HCPT@CD-LHNPs) were prepared using a co-assembly method. At 7 mL/min, HCPT was dispersed in 10 mL of THF containing 10 mg lignin, ultrasonicated for 2 min, stirred at 1000 rpm, and then added to 40 mL of distilled water. During this process of self-assembly, the CD-EHL encapsulated the HCPT into CD-LHNPs. After stirring for 24 h, the suspension was transferred to a dialysis bag for 24 h (water replaced once) to remove the THF and other free molecules.

The suspension of drug-loaded nanoparticles was centrifuged at 11,000 rpm for 15 min, and the absorbance of the supernatant was measured at 254 nm with a UV-Vis spectrophotometer to determine the drug concentration. The absorbance of blank nanoparticles was used for comparison to calculate drug loading.

The drug loading and encapsulation efficiency were calculated according to the following equations:

Drug Loading (%) = (weight of loaded HCPT/weight of HCPT@CD-LHNPs) × 100

Encapsulation Efficiency (%) = (weight of loaded HCPT/weight of initial amount of HCPT) × 100.

### 2.6. In Vitro Release Studies

The *in vitro* release of HCPT from CD-LHNPs was investigated by dialysis of the particles against phosphate-buffered saline (PBS; pH 7.4 and 5.5). In brief, 10 mg of HCPT@LHNPs and 10 mg of HCPT@CD-LHNPs were immersed in 50 mL PBS and then agitated at 100 rpm (temperature: 37 °C). At scheduled intervals, 5 mL was collected from the medium and replaced with the same amount of fresh medium. The samples were then centrifuged at 11,000 rpm for 5 min, and the absorbance (i.e., amount of HCPT released) was measured at 254 nm with a UV-Vis spectrophotometer. The average was determined from the calculated values of at least three replicates. The release rate of the drug was obtained by plotting the data showing the change in absorbance intensity.

### 2.7. In Vitro Cytotoxicity Studies

The cytotoxicity of CD-LHNPs was evaluated using MTT assay. HeLa cells were cultured in a 96-well flat-bottom plate with 6 × 10^3^ cells per well. After incubation for 24 h, different concentrations of HCPT, CD-LHNPs, or HCPT@CD-LHNPs were added to each well, and a powerful magnetic field was established at the periphery of each well. After incubation for 48 h, the cells were washed twice with PBS and then incubated for an additional 2 d at 37 °C in fresh media. Thereafter, the media was removed, and the cells were resuspended in DMSO. The absorbance of each well was measured at 570 nm with a microplate reader.

## 3. Results and Discussion

### 3.1. Characterization of β-Cyclodextrin-Modified EHL

**Figure 1:**
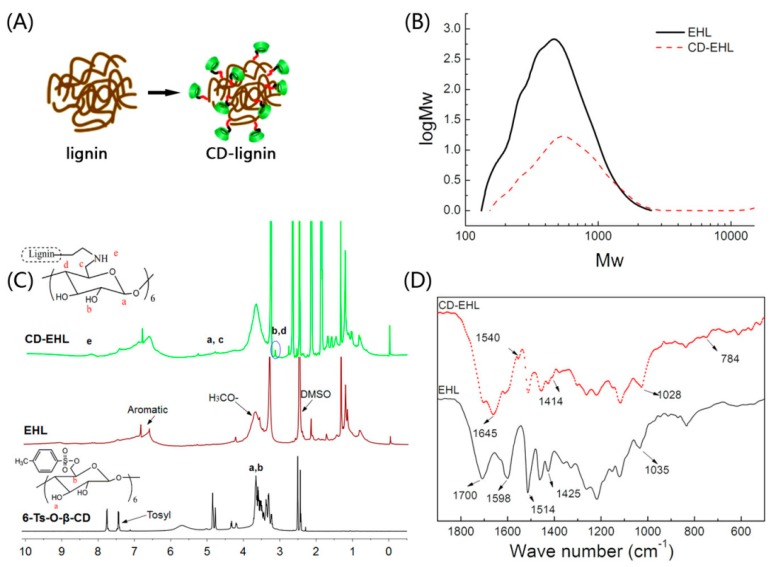
Preparation and characterization of cyclodextrin-(CD) enzymatic-hydrolysis lignin (EHL) (CD-EHL): (**A**) Schematic diagram of β-CD conjugated with EHL; (**B**) molecular weight distribution of EHL and CD-EHL; (**C**) 1H-NMR spectra of 6-TsO-β-CD, EHL and CD-EHL; (**D**) infrared spectrograms of EHL and CD-EHL.

In this study, enzymatic-hydrolysis lignin (EHL) was modified to improve its network structure. The EHL was conjugated with β-cyclodextrin (β-CD) to increase the molecular weight of EHL, as shown in Figure 1A. Gel permeation chromatography was used to measure the molecular weight distribution of the prepared CD-EHL. As illustrated in Figure 1B, when EHL was modified by β-CD, the weight-average molecular weight increased from 500 g/mol to 700 g/mol. The dispersity (PDI) of EHL was 1.34 and that of CD-EHL was 1.57, indicating a high ratio of molecular weight of the modified material.

Fourier transform infrared spectra (Thermo Fisher Nicolet iS10, Waltham, MA, USA) and 1H NMR spectra (Bruker Avance III 500 MHz, Billerica, MA, USA) were determined and exhibited the modification of EHL. In Figure 1C, the 1H NMR spectrum of CD-EHL represents the hydrogen protons in a benzene ring of lignin (6.76–7.72 ppm), methoxyl protons (3.84 ppm), and aliphatic side chains related to a benzene ring (2.6–0.7 ppm) [19], as well as signal for β-CD protons (3.42–3.19 and 4.86–4.42 ppm) [20,21]. More importantly, a secondary amine signal is apparent at 8.22 ppm, indicating a substitution reaction between β-CD and EHL-containing amino. The sulfonyl signal disappeared in the range of 7.75–7.43 ppm, confirming that the sample did not contain residual β-CD.

In Figure 1D, the absorption peak near 1700 cm^−1^ (C=O) corresponds to the C=O stretching vibration for EHL [22]. The absorption peaks at 1598 cm^−1^, 1514 cm^−1^ (C–C), and 1425 cm^−1^ (C–H in the benzene ring skeleton) reflect the stretching vibration of the benzene ring [23,24,25]. Compared with the infrared spectrogram for EHL, N–H and C–N stretching vibration bands appeared at 1645 cm^−1^ and 1540 cm^−1^, respectively [26]. Furthermore, due to modification by β-CD, an O–H stretching vibration band appeared in β-CD, near 1414 cm^−1^ in CD-EHL, and the stretching vibration of C=O shifted from 1035 cm^−1^ to 1028 cm^−1^ [27,28]. The above patterns indicate the occurrence of a Hinsberg reaction between β-CD and EHL, and successful conjugation of β-CD with EHL.

### 3.2. Morphology, Size, and Chemical Characterization of β-CD-Modified LHNPs

**Figure 2:**
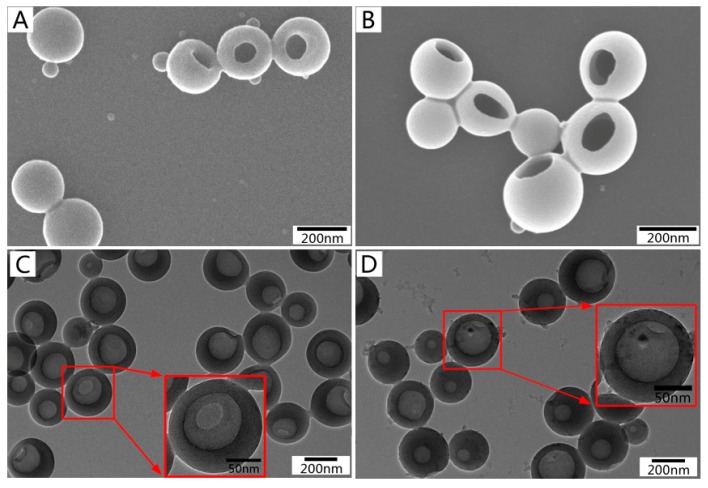
SEM images of (**A**) lignin-based hollow nanoparticles (LHNPs) and (**B**) CD-LHNPs, and TEM images of (**C**) LHNPs, and (**D**) CD-LHNPs.

The suspension of lignin hollow nanoparticles was dropped onto silicon wafers and copper grids support film, respectively. It was then dried at room temperature for 24 h. The morphology of the particles was observed by SEM (S-4800 scanning electron microscope, Hitachi, Tokyo, Japan) and TEM (JEM-2100 transmission electron microscope, JEOL, Tokyo, Japan), respectively. The images from the fabrication of EHL hollow nanoparticles (HNPs) and CD-LHNPs, were obtained and are presented in Figure 2. Although the self-assembly of EHL into nanoparticles is similar to that of other amphipathic polymer colloids, these nanoparticles exhibit specific characteristics. The SEM images of LHNPs and CD-LHNPs show the spherical hollow structure with a single hole on the surface of the particles. The TEM images displayed a clear contrast between the center and the shell, which further supported the hollowness of the particles. And the figure shows that the outer layer of CD-LHNPs was rougher than that of LHNPs, which may be related to the increased β-CD monomers on the outer structure (The absence of this phenomenon in SEM images is due to the gold spraying process of the sample before testing).

**Figure 3:**
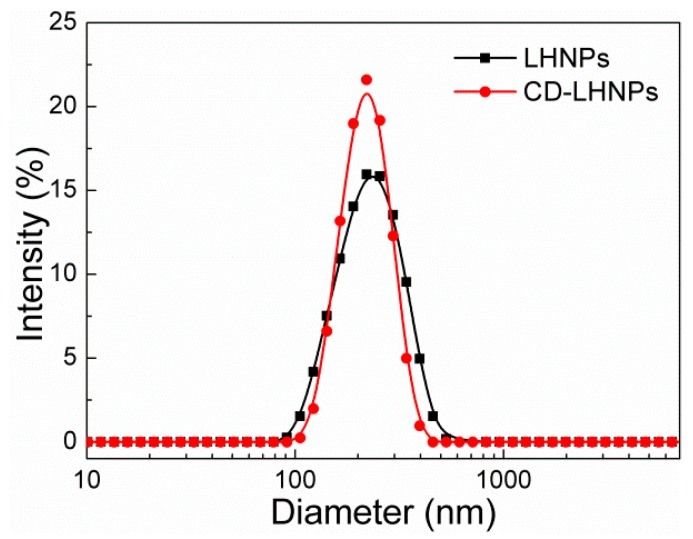
Size distribution of LHNPs and CD-LHNPs by dynamic light scattering (DLS).

Dynamic light scattering (DLS) revealed differences in self-assembly between the two kinds of lignin-based nanoparticles. As illustrated in Figure 3, the average diameters for LHNPs and CD-LHNPs were about 200 nm, respectively, consistent with the TEM results. There was a narrow size distribution in solution, indicating relatively stable particle size, likely attributable to the EHL’s small PDI. The results confirmed that the modified lignin can still self-assemble into nanoparticles.

To study the hydrophilic performance of lignin-based nanoparticles, the static contact angles of membranes formed by the two kinds of nanoparticles suspension on gold sheet were measured (concentration: 5 mg/mL). As shown in Figure S1, the contact angle of CD-LHNP (3.43°) was significantly lower than that of LHNP (80.9°). It indicated that the surface wettability of CD-LHNP with the gold sheet was better, which was favorable for spreading on the surface of the gold substrate. Tetrahydrofuran was used as a good solvent and water was used as a poor solvent to prepare the nanoparticles so hydrophilic chains of lignin would aggregate on the nanoparticles’ shell due to the hydrophobic effect [29]. When β-cyclodextrin was conjugated with EHL, the ratio of hydrophilic groups on the molecular chain was increased, leading to a lower surface contact angle of CD-LHNPs after self-assembly.

**Figure 4:**
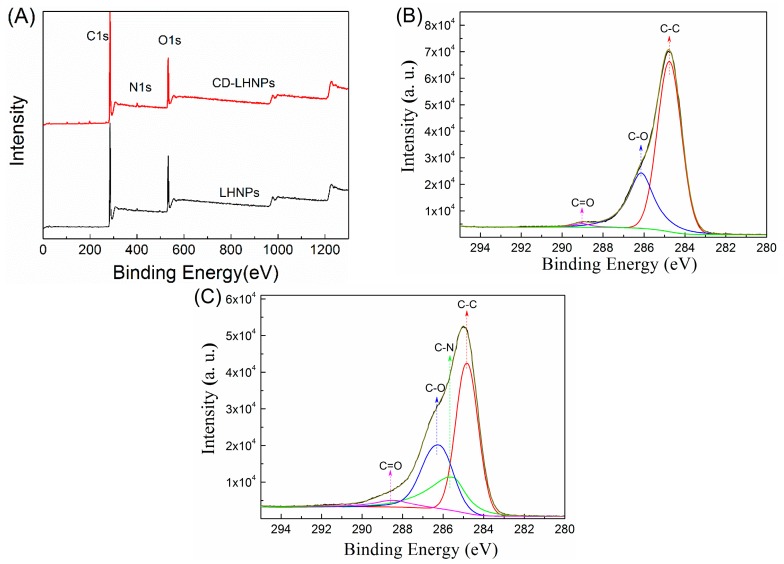
XPS scans of LHNPs and CD-LHNPs. (**A**) XPS wide scans of the LHNPs and CD-LHNPs, (**B**) deconvolution of C1s signals for the LHNPs and (**C**) deconvolution of C1s signals for the CD-LHNPs.

To investigate the surface composition of HNPs, the elemental composition for the top 1−5 nm of depth was analyzed by X-ray photoelectron spectroscopy (XPS; Axis Ultra, Kratos, England), as shown in Figure 4 and Table 1. Aromatic rings compose the main hydrophobic groups in lignin, and a higher C content leads to a stronger hydrophobicity [30]. As shown in Table 1, the content of C decreased in CD-LHNPs, consistent with fewer surface-region hydrophobic chains. Increased N content was caused by the introduction of amino groups during the experiment. Because the hydrophilic groups in lignin are mainly hydroxyl and carboxyl groups, a higher O/C will lead to higher hydrophilicity in the shell’s outer layer [31]. The O/C ratio for CD-LHNP’s surface regions was 0.23%, higher than the 0.19% obtained for LHNPs and consistent with having a more hydrophilic outer surface for CD-LHNPs compared to that of LHNPs.

The C1s XPS spectra for lignin-based nanoparticles are shown in Figure 4B,C. After the grafting of β-cyclodextrin onto EHL, a new peak corresponding to the carbon atoms for C–N (C1s at 285.52 eV) was observed. As shown in Table 1, the proportion of carbon without oxidization (C–C, C–H; 284.8 eV) decreased from 68.82% (LHNPs) to 43.93% (CD-LHNPs). At the same time, the alcohol content (C–OH, 286.14 eV) increased from 29.55% (LHNPs) to 31.23% (CD-LHNPs). Accordingly, compared with LHNPs, the surface of the CD-LHNPs is more hydrophilic, consistent with the contact angle test results.

### 3.3. Space Structure Performance Characteristics of LHNPs

**Figure 5:**
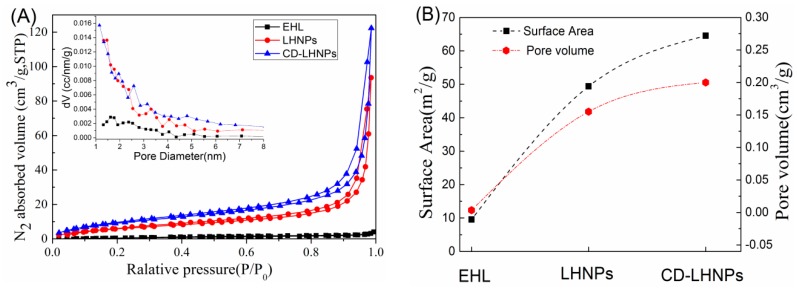
(**A**) Nitrogen sorption isotherms of EHL, LHNPs and CD-LHNPs; (**B**) surface area and pore volume.

Nitrogen adsorption–desorption curves of EHL, LHNPs, and CD-LHNPs were determined and are shown in Figure 5A. The inset shows the pore size distribution as measured by DFT. The curve type is Brunauer IV [32]. The pore size ranged between 1 nm and 6 nm, indicating a microporous or mesoporous material. The specific surface areas and pore volumes for EHL and LHNPs were determined and are shown in Figure 5B. After self-assembly into particles, the specific surface area and pore volume increased significantly. The specific surface area and pore volume in CD-LHNPs were 64.51 m^2^ g^−1^ and 0.20 cm^3^ g^−1^, respectively, while those of LHNPs were 49.37 m^2^ g^−1^ and 0.155 cm^3^ g^−1^, respectively. Nanospheres of β-CD-modified EHL show 23% and 22% higher values than those prepared from pure EHL, indicating that β-CD significantly expands the spatial network in LHNPs.

To further evaluate the stability of LHNPs under physiological pH, changes of average size, PDI, and zeta potential were measured in a phosphate-buffered saline (PBS; pH 7.4) after incubation at 37 °C for 6 days. The average sizes of the two kinds of nanoparticles remained constant during incubation (Figure S2A). As shown in Figure S2B, the PDI value of CD-LHNPs remained stable, and that of LHNPs changed slightly. Slight fluctuations in zeta potential were observed over time (Figure S2C), which may suggest that PBS was adsorbed by surface nanoparticles. This result indicates that LHNPs have high colloidal stability in PBS, without aggregation, for up to 6 days.

### 3.4. Encapsulation and Sustained-Release Capacity of CD-LHNPs

Table 2 shows the measured encapsulation efficiency (EE) and drug loading (DL) for the two nanoparticles. Lignin-based nanoparticles show good drug encapsulation capabilities for the hydrophobic camptothecin, and drug loading increased with drug content. For *m*_0_/*m* of about 40%, the drug loading and encapsulation efficiency were relatively high. The loading efficiency of CD-LHNPs was greater than that of LHNPs because the specific surface area and the porosity values for CD-LHNPs were larger than those of LHNPs.

**Figure 6:**
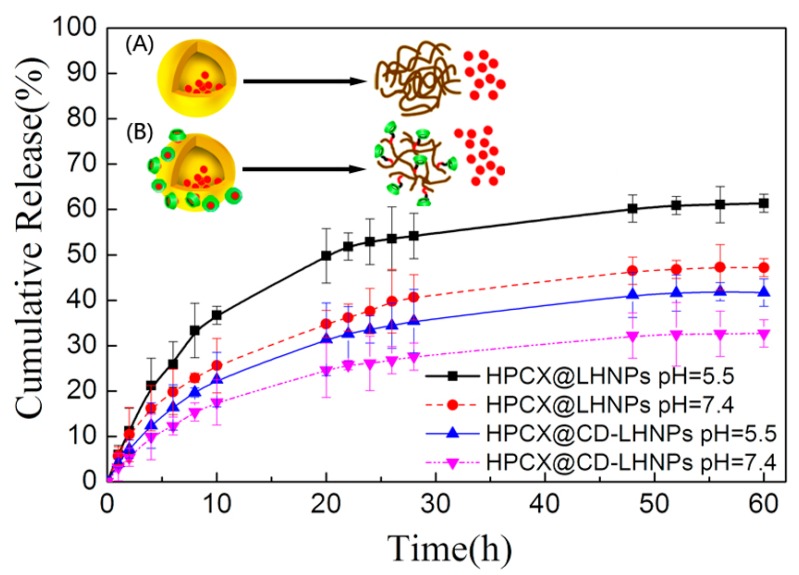
Release profiles of HCPT from HCPT@LHNPs and HCPT@CD-LHNPs. The inset shows a schematic illustration of drug release for (**A**) HCPT@LHNPs and (**B**) HCPT@CD-LHNPs.

To test the ability of these particles to function in more physiological conditions, we simulated a tumor or intracellular microenvironment (pH: 5.5) and a physiological (pH: 7.4) environment. Two buffer solutions were selected as release media, enabling the evaluation of drug release at 37 °C from HCPT@LHNPs and HCPT@CD-LHNPs. As shown in Figure 6, the release curves for the two kinds of nanoparticles show significant sustained-release potential. HCPT@CD-LHNPs possessed more β-CD molecules with specific sites interacting with HCPT, resulting in a lower burst release (insert B in Figure 6). At pH 7.4, the accumulated release rates of HCPT@LHNPs and HCPT@CD-LHNPs after 60 h were 47.2% and 32.7%, respectively. Under acidic conditions, 61.4% and 41.7% HCPT releases were obtained. This result indicated that at pH 7.4, the two nanoparticles show slower HCPT release rates. Overall, although both kinds of nanoparticles can control the release of HCPT, the synergistic effect of HCPT@CD-LHNPs was superior to that of LHNPs due to the use of β-CD.

### 3.5. In Vitro Cytotoxicity Studies

**Figure 7:**
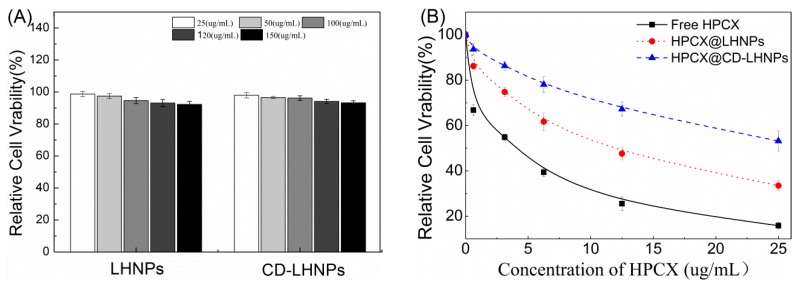
Viability of HeLa cells after 48 h incubation at 37 °C. (**A**) LHNPs and CD-LHNPs at different concentrations; (**B**) Free HCPT, HCPT@LHNPs and HCPT@CD-LHNPs.

The data in Figure 7A illustrate that when the concentration of particles was increased to 150 μg/mL, the survival rate of HeLa cells after 48 h was still higher than 95%. Overall, cyclodextrin was demonstrated to be an oligosaccharide with excellent biocompatibility and biodegradability. The results suggested that LHNPs would not impact the growth of HeLa cells.

As shown in Figure 7B, free HCPT, HCPT@LHNPs, and HCPT@CD-LHNPs were evaluated for toxicity against HeLa cells. The results indicated that free HCPT and drug-loaded nanoparticles both inhibit the growth of HeLa cells. The survival rate of cells increased as the HCPT concentration decreased. However, compared with free HCPT, the toxicity of HCPT-loaded nanoparticles was significantly reduced. The small, free molecules passed through cell membranes more easily and entered the cells. The drug-loaded nanoparticles were taken up by cells and then the drug had to be released from the particles to have an effect. With their superior sustained-release capacity compared to LHNPs, the toxicity of CD-LHNPs in HCPT@CD-LHNPs was lower, at the same incubation time and concentration, than that of LHNPs in HCPT@LHNPs. This result was consistent with that of the release curve.

## 4. Conclusions

In this study, silanization, sulfonylation, and molecular self-assembly were used to prepare lignin-based nanoparticles (LHNPs and CD-LHNPs) with efficient encapsulation and good capacity for sustained-release medication delivery. The size of the nanoparticles was about 200 nm, and the structure and performance of the prepared particles remained stable in PBS (pH: 7.4) for 6 days.

CD-EHL is formed by conjugating β-cyclodextrin (β-CD) with enzymatic-hydrolysis lignin (EHL). The prepared CD-LHNPs exhibited a higher specific surface area and porosity and better encapsulation capability compared to LHNPs.

Compared to LHNPs, more specific sites of CD-LHNPs interact with drug molecules, resulting in good sustained-release capacity. The experimental results show that the conjugation of β-cyclodextrin does not alter the low cytotoxicity of lignin nanoparticles. Additionally, the enhanced hydrophilic surface promotes the circulation of nanoparticles in blood.

## Figures and Tables

**Table 1:** Elemental distribution of LHNPs and CD-LHNPs, and their outer surfaces.

Samples	Surface Elemental Composition (%)							
	C%	O%	N%	O/C Ratio	C–C	C–N	C–O	C=O
LHNPs	82.96	15.9	1.13	0.19	68. 82	0	29.55	1.64
CD-LHNPs	78.62	18.7	2.69	0.23	43. 93	20.96	31.23	3.88

**Table 2:** Variation of drug loading and encapsulation in LHNPs and CD-LHNPs with *m*_0_/*m*.

Sample	LHNPs		CD-LHNPs	
*m*_0_/*m*	EE (%)	DL (%)	EE (%)	DL (%)
10%	58.6 ± 5	5.54 ± 0.3	82.4 ± 3	7.61 ± 1
20%	59.4 ± 7	10.62 ± 0.7	78.5 ± 5	13.57 ± 2
40%	51.1 ± 7	16.97 ± 0.9	70.6 ± 9	22.02 ± 2
60%	40.5 ± 11	19.55 ± 0.7	54.8 ± 9	24.76 ± 2

Note: *m*_0_ is the mass of hydroxycamptothecin (HCPT) (mg), *m* is the mass of EHL (mg).

## Supplementary Materials

The following are available online at https://www.mdpi.com/2079-4991/9/7/997/s1, Figure S1: Surface water contact angle of the films prepared with LHNPs and CD-LHNPs. Figure S2: The stability of LHNPs and CD-LHNPs in PBS (pH 7.4) at 37 °C. (A) Average size, (B) PDI, (C) Zeta.

## References

[1] Ding Z., Li F., Wen J.L., Wang X., Sun R.C. (2018). Gram-scale synthesis of single-crystalline graphene quantum dots derived from lignin biomass. Green Chem..

[2] Mariarosaria T., Francesca C., Pasquale M., Flavia C., Federica M., Claudia C. (2014). Ultrasound driven assembly of lignin into microcapsules for storage and delivery of hydrophobic molecules. Biomacromolecules.

[3] Li X., Li M., Pu Y., Ragauskas A.J., Klett A.S., Thies M., Zheng Y. (2018). Inhibitory Effects of Lignin on Enzymatic Hydrolysis: The Role of Lignin Chemistry and Molecular Weight. Renew. Energy.

[4] Cree I.A., Charlton P. (2017). Molecular chess? Hallmarks of anti-cancer drug resistance. BMC Cancer.

[5] Shaffer S.M., Dunagin M.C., Torborg S.R., Torre E.A., Emert B., Krepler C., Beqiri M., Sproesser K., Brafford P.A., Xiao M. (2017). Rare cell variability and drug-induced reprogramming as a mode of cancer drug resistance. Nature.

[6] Kamran M., Long Z.J., Xu D., Lv S.S., Liu B., Wang C.L., Xu J., Lam E.W., Liu Q. (2017). Aurora kinase A regulates Survivin stability through targeting FBXL7 in gastric cancer drug resistance and prognosis. Oncogenesis.

[7] Marzi L., Agama K., Murai J., Difilippantonio S., James A., Peer C.J., Figg W.D., Beck D., Elsayed M., Cushman M. (2018). Novel fluoroindenoisoquinoline non-camptothecin topoisomerase I inhibitors. Mol. Cancer Ther..

[8] Tian X., Nguyen M., Foote H.P., Caster J.M., Roche K.C., Peters C.G., Wu P., Jayaraman L., Garmey E.G., Tepper J.E. (2017). CRLX101, a Nanoparticle-Drug Conjugate Containing Camptothecin, Improves Rectal Cancer Chemoradiotherapy by Inhibiting DNA Repair and HIF1α. Cancer Res..

[9] Tardy B.L., Richardson J.J., Guo J., Lehtonen J., Rojas O.J. (2018). Lignin Nano- and Microparticles as Template for Nanostructured Materials: Formation of Hollow Metal-Phenolic Capsules. Green Chem..

[10] Zelepukin I.V., Yaremenko A.V., Shipunova V.O., Babenyshev A., Nikitin M. (2018). Nanoparticle-based drug delivery: Via RBC-hitchhiking for the inhibition of lung metastases growth. Nanoscale.

[11] Ejima H., Richardson J.J., Caruso F. (2017). Metal-phenolic networks as a versatile platform to engineer nanomaterials and biointerfaces. Nano Today.

[12] Lin D., Zhu W., Rui L., Si C. (2018). Lignin-Containing Self-Nanoemulsifying Drug Delivery System for Enhance Stability and Oral Absorption of trans-Resveratrol. Part. Part. Syst. Charact..

[13] Chen N., Dempere L.A., Tong Z. (2016). Synthesis of pH-responsive Lignin Based Nanocapsules for Controlled Release of Hydrophobic Molecules. ACS Sustain. Chem..

[14] Li Y., Zhou M., Pang Y., Qiu X. (2017). Lignin-Based Microsphere: Preparation and Performance on Encapsulating the Pesticide Avermectin. ACS Sustain. Chem. Eng..

[15] Dai L., Liu R., Hu L., Zou Z., Si C. (2017). Lignin nanoparticle as a novel green carrier for the efficient delivery of resveratrol. ACS Sustain. Chem. Eng..

[16] Marcelo G., López-González M., Trabado I., Rodrigo M.M., Valiente M., Mendicuti F. (2016). Lignin inspired PEG hydrogels for drug delivery. Mater. Today Commun..

[17] Cheng J.G., Zhang Y.M., Yu L. (2018). Supramolecular Assembly of Thiolated Cyclodextrin and Ferrocene Derivative for Controlled Drug Delivery. Chemnanomat.

[18] Sonia M., Cristina M., Kostas K., Maurizio P., Ester V. (2015). Nanocomposite Hydrogels: 3D Polymer-Nanoparticle Synergies for On-Demand Drug Delivery. ACS Nano.

[19] Qiu H.B., Yang S., Han Y., Shen X.S., Fan D., Li G., Chu F. (2018). Improvement of the Performance of Plantation Wood by Grafting Water-Soluble Vinyl Monomers onto Cell Walls. ACS Sustain. Chem. Eng..

[20] Lu B., Wei L., Meng G., Hou J., Liu Z., Guo X. (2017). Synthesis of Self-assemble pH-responsive Cyclodextrin Block Copolymer for Sustained Anticancer Drug Delivery. Chin. J. Poly. Sci..

[21] Liu M., Lv P., Liao R., Zhao Y., Bo Y. (2017). Synthesis, characterization and biological activity of Rhein-cyclodextrin conjugate. J. Mol. Struct..

[22] Xiong F., Chu F., Li G., Wang S., Qin T., Han Y., Chen Y. (2017). Preparation and formation mechanism of size-controlled lignin nanospheres by self-assembly. Ind. Crops Prod..

[23] Casas A., Alonso M.V., Oliet M., Rojo E., Rodríguez F. (2012). FTIR analysis of lignin regenerated from Pinus radiata and Eucalyptus globulus woods dissolved in imidazolium-based ionic liquids. J. Chem. Technol. Biotechnol..

[24] Liu X., Wang J., Li S., Zhuang X., Xu Y., Wang C., Chu F. (2014). Preparation and properties of UV-absorbent lignin graft copolymer films from lignocellulosic butanol residue. Ind. Crops Prod..

[25] Xiong F., Han Y., Wang S., Li G., Qin T., Chen Y., Chu F. (2017). Preparation and Formation Mechanism of Renewable Lignin Hollow Nanospheres with a Single Hole by Self-Assembly. ACS Sustain. Chem. Eng..

[26] Xiong F., Han Y., Li G., Qin T., Wang S., Chu F. (2016). Synthesis and characterization of renewable woody nanoparticles fluorescently labeled by pyrene. Ind. Crops Prod..

[27] Cao J., Xiao G., Xu X., Shen D., Jin B. (2013). Study on carbonization of lignin by TG-FTIR and high-temperature;carbonization reactor. Fuel Process. Technol..

[28] Li Z., Bo Z., Jia S., Ma M., Hao J. (2017). Novel supramolecular organogel based on β-cyclodextrin as a green drug carrier for enhancing anticancer effects. J. Mol. Liquids.

[29] Hong N., Yuan L., Qiu X. (2016). A highly efficient dispersant from black liquor for carbendazim suspension concentrate: Preparation, self-assembly behavior and investigation of dispersion mechanism. J. Appl. Polym. Sci..

[30] Hong N., Wei Y., Xue Y., Zeng W., Huang J., Xie W., Qiu X., Yuan L. (2016). A novel and highly efficient polymerization of sulfomethylated alkaline lignins via alkyl chain cross-linking method. Holzforschung.

[31] Yong Q., Deng Y., Qiu X., Hao L., Yang D. (2014). Formation of uniform colloidal spheres from lignin, a renewable resource recovered from pulping spent liquor. Green Chem..

[32] Lunde P.J., Kester F.L. (1975). Chemical and physical gas adsorption in finite multimolecular layers. Chem. Eng. Sci..

