# Supplementary material for: Lignin-Based Hollow Nanoparticles for Controlled Drug Delivery: Grafting Preparation Using β-Cyclodextrin/Enzymatic-Hydrolysis Lignin
Source: Nanomaterials (Basel). 2019 Jul 11;9(7):997. doi: 10.3390/nano9070997 (PMC6669448; doi:10.3390/nano9070997)
Supplement: Supplementary file 1 [file nanomaterials-09-00997-s001.pdf]

Supplementary information

Lignin-Based Hollow Nanoparticles for Controlled Drug Delivery: Grafting Preparation Using β-Cyclodextrin/Enzymatic-Hydrolysis Lignin

Yu Zhou [1,2], Yanming Ha [1,*], Gaiyun Li [1], Sheng Yang [1] and Fuxiang Chu [1,*]

[1] Research Institute of Wood Industry, Chinese Academy of Forestry, Xiangshan Road, Beijing, 100089, China; zhouyu_sky@126.com (Y.Z.); ligy@caf.ac.cn (G.L.); yangsheng@criwi.org.cn (S.Y.)

[2] School of Chemistry and Chemical Engineering, Yancheng Institute of Technology, Yancheng City, 224051, China

[*] Corresponding author: hanym@caf.ac.cn (Y.H.); chufuxiang@caf.ac.cn (F.C.); Tel.: +86-10-62889433 (Y.H. & F.C.); Fax: +86-10-62889433 (Y.H. & F.C.)

The number of pages: 3

The number of figures: 2

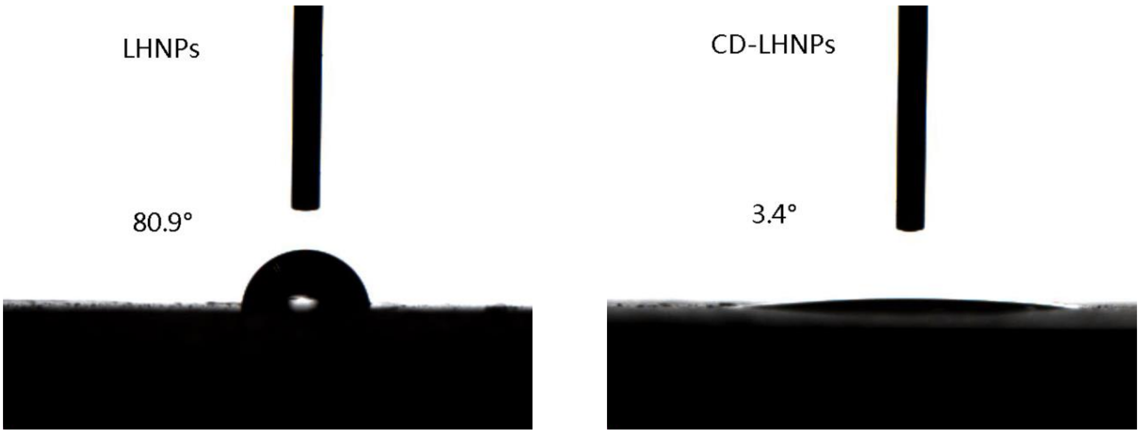

Figure S1. Surface water contact angle of the films prepared with LHNPs and CD-LHNPs.

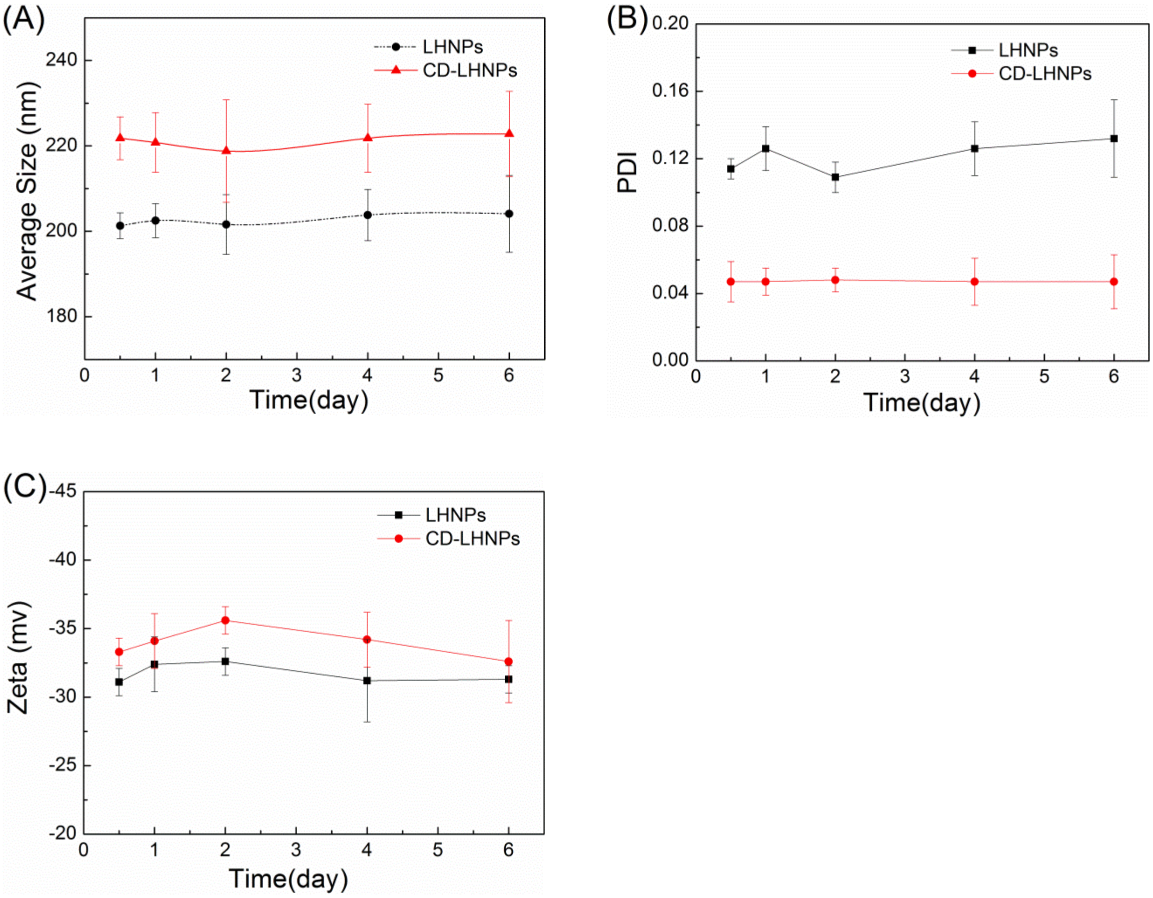

Figure S2. The stability of LHNPs and CD-LHNPs in PBS (pH 7.4) at 37 °C. (**A**) Average size, (**B**) PDI, and (**C**) Zeta.
